# Infantile Paralysis

**Published:** 1873-09

**Authors:** 


### Infantile Paralysis.

In his very able work on "Localized Electrization and its application to Pathology and Therapeutics," Dr. Duchenne de Boulogne makes some valuable remarks on infantile paralysis.

In the chapter entitled "Atrophic Paralysis of Childhood," the author gives a finished study of this affection by adding to his own work the results obtained by Laborde, Cornil, Duchenne (fils), Prevost and Vulpian, Lockhart Clarke, Charcot and Joffroy, Roger and Damaschino.

The disease, which is generally announced by fever, may, contrary to the opinion maintained by Roger and Laborde, be in some cases entirely apyretic.

Soon, as is well known, a great number of muscles are paralysed, and the author remarks that the electro-muscular contractility disappears on the seventh day.

This point is very important, for the paralysed muscles which at the end of this time preserve a part of this contractility are not slow in recovering their motility, and that the more quickly as the irritability is less weakened.

The works of the authors above cited have established in a solid manner that the atrophic paralysis of childhood is a particular disease of the spinal marrow, but whilst some make out a sort of diffused myelitis without precise localization, MM. Charcot and Joffroy have above all sought to show that the lesion is primitively circumscribed to the cells of the anterior regions of the cord. The facts observed by MM. Roger and Damaschino were contrary to this hypothesis, and in the presence of this difficulty Dr Duchenne pronounces no opinion.

The rules of treatment are very simple. At first antiphlogistics but no electrization. At the end of three or four weeks one practises localized muscular excitation, for the double purpose of remedying atrophy and preventing deformities. Continuous currents are not borne by children on account of the pain produced at the point of application of the rheophores.

It is habitually believed that the disease of which the symptomatic whole is now designated under the name atrophic paralysis of childhood is special to that age. Dr. Duchenne de Boulogne meanwhile has equally observed it in the adult, and he proposes to designate this variety under the name of *acute anterior spinal paralysis of the adult* (by atrophy of the anterior cells of the cord). Clinical observations show that the disease is the same as in the child; but the anatomy is at fault.

It should be mentioned that for the most part Duchenne adheres to his views as to the superiority of Faradic electrization (or the induced current). He is thus in opposition to Remak, who prefers the continuous current.

In a communication to the Société de Biologie, M. Vulpain remarks that to explain the rapid loss of muscular contractility in infantile paralysis, he imagines that a degeneration analogous to that which results from section of these nerves occurs in the nerves supplying the nerves which become atrophied. The phlegmasic process which attends the febrile disturbance, so frequent at the beginning of the disease, and which arises in the grey substance of the cord, destroys the normal relations between the nervous fibres and the cells, and thus strikes with degeneration the nervous cords deprived of their trophic centres

If one finds after death only a simple atrophy, with preservation of the nervous tubes which have become more or less thinned in the anterior roots and the motor nerves, it is because the autopsies were made at a very advanced period of the disease when an autogenic restoration has been produced in the nervous threads primitively attacked with granular degeneration.

In progressive muscular paralysis lesions of the grey substance occur slowly and less rapidly destroy the connexions between the nervous fibres and their cells, and are only followed after the lapse of some time by the loss of muscular contractility.—*Gaz. Med.*, p. 9, 1873.—*Obstetrical Journal, Great Britain and Ireland.*

